# Anti-Obesity Effects of *Tisochrysis lutea* Powder in High-Fat Diet-Induced Obese Mice Through the Regulation of Adipogenesis and Lipid Metabolism

**DOI:** 10.3390/ijms27104277

**Published:** 2026-05-11

**Authors:** Jae-In Eom, Se-Min Kim, Joo Young Lee, Ji-Woo Kim, Dae Yoon Kim, Jae Kwon Lee, Cheol-Ho Pan

**Affiliations:** 1 Microalgae Ask Us Co., Ltd., Gangneung 25441, Republic of Korea; umjaein@maus2020.com (J.-I.E.); kimsemin@maus2020.com (S.-M.K.); jyoung@maus2020.com (J.Y.L.); kimjiwoo@maus2020.com (J.-W.K.); 2 Department of Biology Education, College of Education, Chungbuk National University, Cheongju 28644, Republic of Korea; lnbsky@naver.com

**Keywords:** *Tisochrysis lutea*, microalgal powder, fucoxanthin, DHA, obesity, adipogenesis, lipid metabolism

## Abstract

Obesity is associated with excessive lipid deposition in adipose tissue and the liver, leading to systemic metabolic disturbances. In this study, we investigated the anti-obesity efficacy of *Tisochrysis lutea* (TL) powder, standardized to fucoxanthin (12.18 ± 0.21 mg/g DW) and docosahexaenoic acid (DHA) (16.03 ± 0.49 mg/g DW), in a high-fat diet (HFD)-induced obesity model in C57BL/6N mice. TL supplementation (50–150 mg/kg) over eight weeks significantly reduced body weight gain by up to 63.2%, total white adipose tissue mass by 53.4%, and liver weight by 38.2% compared to the HFD control, without affecting renal safety markers. Histological examination revealed smaller adipocytes and diminished hepatic steatosis in TL-treated groups. Serum triglycerides and leptin concentrations were significantly lowered by 38.5% and 70.1%, respectively, while HFD-induced elevations of ALT and AST were reduced by 61.7% and 38.6%, respectively. At the transcriptional level, TL downregulated adipogenic markers including PPARγ, C/EBPα, and SREBP-1c by 46~54%, as well as lipogenic regulators including FAS and ACC1 by up to 72%. Furthermore, TL treatment upregulated the mRNA levels of HSL and AMPK 2.3- and 2.1-fold, respectively, compared to the HFD control. These findings indicate that fucoxanthin- and DHA-enriched TL powder improves obesity-related metabolic alterations by modulating lipid storage and utilization pathways, supporting its development as a marine-derived functional ingredient for metabolic health management.

## 1. Introduction

Obesity arises from sustained positive energy balance, resulting in progressive enlargement of adipose depots and ectopic lipid accumulation in metabolic organs. This condition is strongly linked to insulin resistance, dyslipidemia, and fatty liver pathology [1,2,3,4]. Although pharmacological treatments exist, long-term weight management remains difficult due to tolerability concerns and the necessity for persistent lifestyle changes [5,6]. Consequently, there is growing interest in food-derived and marine-derived functional ingredients capable of modulating adipose tissue biology and systemic lipid metabolism with improved safety profiles [7,8,9].

Microalgae serve as sustainable biofactories producing a variety of bioactive compounds, including proteins, polysaccharides, carotenoids, sterols, and long-chain omega-3 polyunsaturated fatty acids (PUFAs). Their potential as functional food ingredients to support cardiometabolic health has been increasingly recognized [7,8,9,10,11,12]. Recent reviews highlight that microalgal products may ameliorate features of metabolic syndrome, including obesity-related conditions, through multi-target mechanisms that involve lipid metabolism, inflammation, oxidative stress, and gut–host interactions [10,11,12,13]. *Tisochrysis lutea* (syn. *Isochrysis affinis galbana*), a widely used haptophyte in aquaculture, stands out as a promising source of valuable bioactives, notably the carotenoid fucoxanthin and omega-3 fatty acids such as DHA [14,15,16,17,18,19]. Advances in processing technologies have further supported *T. lutea* as an attractive feedstock for the co-production of fucoxanthin and DHA, enabling greener extraction and purification methods that facilitate standardization and industrial scalability [14,15,16,17,18].

Fucoxanthin, a marine carotenoid, has demonstrated regulatory effects on adipocyte differentiation, hepatic lipid handling, and energy metabolism in experimental models [20,21,22,23,24,25,26]. These mechanisms involve the inhibition of adipocyte differentiation and fatty acid synthesis through modulation of PPARγ and SREBP-1c activity, along with the enhancement of energy-dissipating pathways, including upregulation of UCP1 expression in white adipose tissue [20,21,22,23,24,25,27,28,29]. In parallel, DHA, a long-chain omega-3 fatty acid, is known to modulate lipid metabolism and adipose tissue remodeling; emerging cellular studies reveal that DHA can influence adipogenic programs, including PPARγ-related pathways, in a context-dependent manner [30,31,32,33]. These complementary bioactivities provide a rationale for investigating microalgal matrices enriched in both compounds [10,11,12,14,15,16,17,18,19].

Although several studies have reported the anti-obesity effects of fucoxanthin-containing seaweed or microalgae, they have predominantly focused on extracts or purified fucoxanthin rather than the whole biomass. In contrast, the present study investigated the effects of the whole *Tisochrysis lutea* powder, which contains a complex matrix of bioactive components including fucoxanthin, lipids, and other metabolites. This compositional complexity may yield synergistic biological effects that differ from those of isolated compounds. Therefore, evaluating the anti-obesity potential of *T. lutea* in its whole-powder form provides meaningful insight into its applicability as a functional ingredient [17]. Therefore, we assessed the impact of *Tisochrysis lutea* (TL) powder on diet-induced obesity in mice using phenotypic, biochemical, and gene expression analyses targeting adipose and hepatic lipid regulatory pathways [2,3].

## 2. Results

### 2.1. Characterization of TL Powder

**Figure 1:**
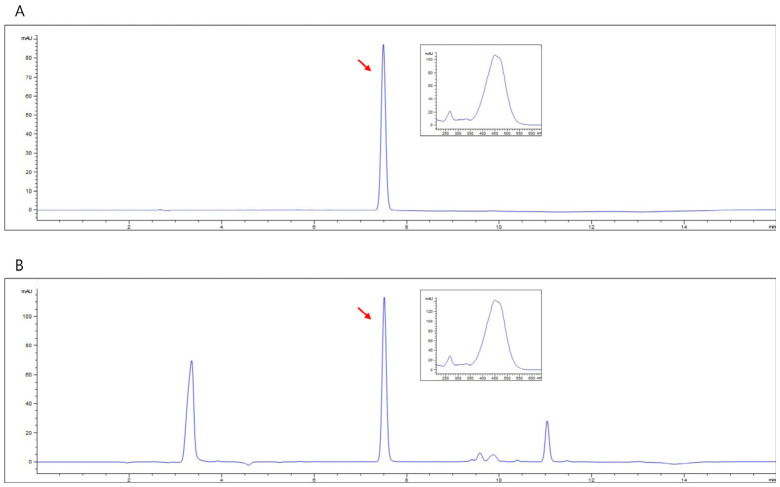
Representative HPLC chromatograms recorded at 450 nm for the fucoxanthin reference standard (**A**) and TL powder extract (**B**). The inset shows the UV–Vis absorption spectrum corresponding to the fucoxanthin peak, and red arrows denote the identified fucoxanthin signals in each chromatogram.

The fatty acid profile of TL powder was determined via gas chromatography coupled with flame ionization detection (GC–FID). The analysis revealed that docosahexaenoic acid (DHA, C22:6n-3), a major omega-3 polyunsaturated fatty acid, accounted for 9.6% of total fatty acids (1603.0 ± 48.5 mg/100 g dry weight), confirming its classification as a DHA-enriched microalgal ingredient (Table 1). Furthermore, fucoxanthin, a characteristic carotenoid, was clearly detected in TL powder via HPLC analysis, as evidenced by a well-resolved peak in its representative chromatogram (Figure 1), and was quantified at 12.18 ± 0.21 mg/g DW.

### 2.2. Impact of TL Powder Supplementation on Body Weight Progression and Feed Efficiency Ratio in Mice with High-Fat-Diet-Induced Obesity

High-fat diet (HFD) feeding significantly increased body weight compared with the normal diet (ND) group. Oral administration of TL powder markedly attenuated HFD-induced weight gain in a dose-dependent manner (Table 2). ND-fed mice gained 9 g (44.3% of initial body weight), whereas HFD-fed mice gained 20.1 g (96.2% of initial body weight). Notably, mice receiving the highest dose of TL powder (150 mg/kg body weight) exhibited a body weight increase of only 7.4 g (35.6% of initial body weight). The feed efficiency ratio (FER) was significantly reduced in TL-treated groups, with a 52.88% decrease observed in the TL150 group.

### 2.3. Impact of TL Powder Supplementation on Adipose Tissue and Liver Weights in HFD-Induced Obesity

As shown in Table 3, TL powder administration significantly reduced total white adipose tissue mass in HFD-fed mice in a dose-dependent manner. Notably, the TL150 group achieved a reduction of over 50% in total white adipose mass. This weight-reducing effect was consistent across all individual fat depots, including epididymal, retroperitoneal, mesenteric, and inguinal adipose tissues (Table 3). Furthermore, TL150 supplementation markedly attenuated hepatic enlargement, resulting in a 38.2% reduction in liver weight compared to the untreated HFD group.

### 2.4. Impact of TL Powder Supplementation on Adipocyte Morphology in Epididymal Adipose Tissue in HFD-Induced Obesity

**Figure 2:**
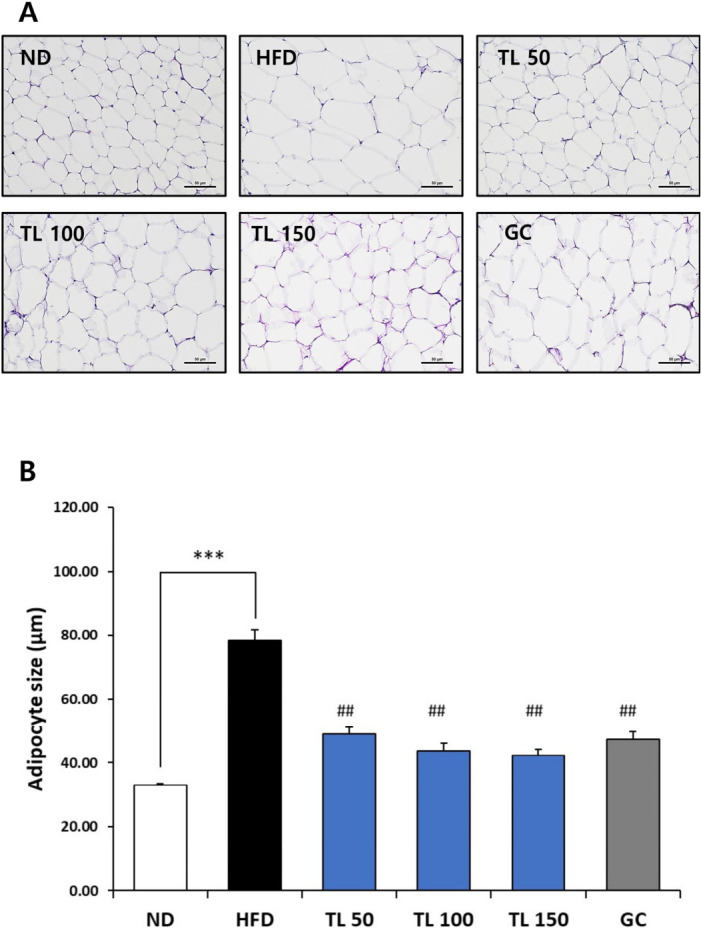
Histological analysis of epididymal adipose tissue in HFD-induced obese mice. (**A**) Representative hematoxylin and eosin (H&E)-stained sections of epididymal adipose tissue from ND, HFD, TL50, TL100, TL150, and GC groups (scale bar = 50 μm). (**B**) Quantitative analysis of adipocyte size measured from H&E-stained sections. Values are expressed as means ± SD (*n* = 5). *** *p* < 0.001 denotes statistical significance relative to the ND group. ^##^ *p* < 0.01 denotes statistical significance relative to the HFD group. ND: normal diet; HFD: high-fat diet; TL50: HFD supplemented with TL powder (50 mg/kg BW); TL100: HFD supplemented with TL powder (100 mg/kg BW); TL150: HFD supplemented with TL powder (150 mg/kg BW); GC: HFD supplemented with *Garcinia cambogia* extract (200 mg/kg BW).

Adipocyte morphology in epididymal fat was assessed via histological examination of hematoxylin and eosin (H&E)-stained tissue sections. As shown in Figure 2, epididymal adipose tissue revealed pronounced adipocyte hypertrophy in HFD-fed mice. In contrast, TL powder treatment significantly reduced adipocyte size and improved adipose tissue morphology, indicating suppression of adipose tissue expansion.

### 2.5. Impact of TL Powder Supplementation on Serum Biochemical Parameters in HFD-Induced Obesity

Serum biochemical analysis was performed to determine whether TL powder altered lipid profiles, liver enzymes, and metabolic parameters in obese mice (Table 4). Blood glucose concentrations were markedly higher in HFD-fed mice (306.6 ± 15.1 mg/dL) than in those maintained on the ND (186.9 ± 13.6 mg/dL; *p* < 0.001), reflecting HFD-associated hyperglycemia. However, no statistically significant differences in blood glucose levels were observed among the HFD, TL50, TL100, TL150, and GC groups. These findings suggest that TL powder and GC do not significantly affect blood glucose levels in HFD-induced obese animals.

Among the lipid profile parameters, triglyceride (TG) levels were elevated in HFD-fed mice. However, TL powder supplementation reduced TG levels in a dose-dependent manner. In particular, the TL150 group exhibited an approximately 38.5% reduction compared with the HFD control group. Although total cholesterol levels tended to decrease in a dose-responsive manner with TL powder supplementation, none of the lipid parameters, including total cholesterol, LDL-cholesterol, and HDL-cholesterol, showed statistically significant changes.

Serum ALT and AST levels, established markers of hepatocellular injury, were significantly elevated in HFD-fed mice. TL powder supplementation mitigated these increases. Conversely, indicators of renal function, such as creatinine and blood urea nitrogen (BUN), remained unchanged following TL powder treatment in obese mice. Collectively, these results indicate that TL powder alleviates obesity-associated lipid dysregulation and hepatic stress without inducing detectable renal or systemic toxicity.

The HFD group showed significantly higher serum leptin levels compared to the ND group (*p* < 0.001). However, TL powder supplementation reduced serum leptin levels in a dose-dependent manner (*p* < 0.001 vs. HFD).

### 2.6. Effects of TL Powder on Hepatic Lipid Accumulation in HFD-Induced Obese Mice

**Figure 3:**
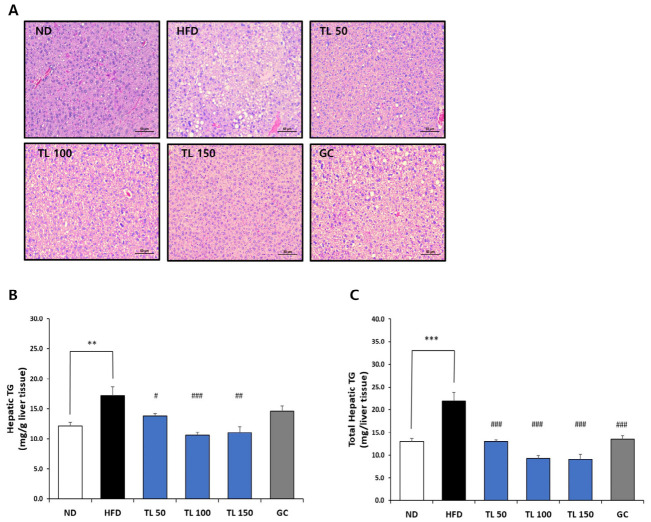
Histological examination of liver and hepatic TG content in HFD-induced obesity. (**A**) Representative H&E-stained liver sections from ND, HFD, TL50, TL100, TL150, and GC groups (scale bar = 50 μm). (**B**) Hepatic TG concentrations expressed as mg per gram of liver tissue and (**C**) total liver TG content (mg per whole liver) were measured and compared across groups. Values are expressed as means ± SD (*n* = 10 mice per group). ** *p* < 0.01 and *** *p* < 0.001 denote statistical significance relative to the ND group. ^#^ *p* < 0.05, ^##^ *p* < 0.01, and ^###^ *p* < 0.001 denote statistical significance relative to the HFD group. ND: normal diet; HFD: high-fat diet; TL50: HFD supplemented with TL powder (50 mg/kg BW); TL100: HFD supplemented with TL powder (100 mg/kg BW); TL150: HFD supplemented with TL powder (150 mg/kg BW); GC: HFD supplemented with *Garcinia cambogia* extract (200 mg/kg BW).

H&E staining revealed prominent lipid deposition in the livers of HFD-fed mice. The hepatic sections from the ND group displayed preserved architecture without detectable fat accumulation, whereas those from the HFD group exhibited pronounced steatosis characterized by extensive intracellular lipid vacuolation (Figure 3A). Notably, lipid accumulation was markedly reduced in all groups treated with TL powder compared with the HFD group, indicating an improvement in hepatic lipid metabolism.

TG levels normalized to liver weight were substantially increased in the HFD group (17.2 ± 1.5 mg/g tissue) relative to the ND group (12.2 ± 0.6 mg/g tissue) (Figure 3B). A comparable pattern was observed for total hepatic TG burden, which was significantly greater in the HFD group (21.8 ± 2.0 mg per liver) than in the ND group (12.9 ± 0.8 mg per liver) (Figure 3C). Administration of TL powder significantly lowered both relative and absolute hepatic TG content compared with the HFD group, reflecting a reduction in liver fat accumulation.

### 2.7. Regulation of Adipogenesis- and Lipid Metabolism-Related Gene Expression via TL Powder in HFD-Induced Obesity

**Figure 4:**
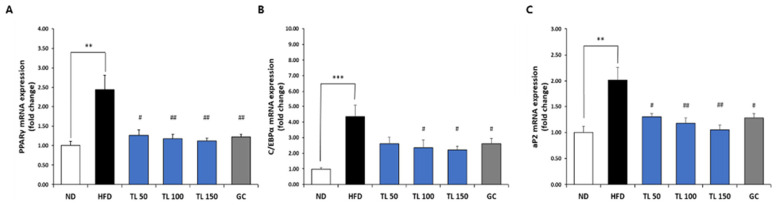
Assessment of adipogenic marker expression in epididymal adipose tissue from mice with HFD–induced obesity. Transcript levels of PPARγ (**A**), C/EBPα (**B**), and aP2 (**C**) were quantified using real-time quantitative PCR with specific primer sets. Expression values were normalized to the housekeeping gene GAPDH and presented as fold differences relative to the ND group. Data are presented as means ± SD (*n* = 7 mice per group). ** *p* < 0.01 and *** *p* < 0.001 denote statistical significance relative to the ND group. ^#^ *p* < 0.05 and ^##^ *p* < 0.01 denote statistical significance relative to the HFD group. ND: normal diet; HFD: high-fat diet; TL50: HFD supplemented with TL powder (50 mg/kg BW); TL100: HFD supplemented with TL powder (100 mg/kg BW); TL150: HFD supplemented with TL powder (150 mg/kg BW); GC: HFD supplemented with *Garcinia cambogia* extract (200 mg/kg BW).

**Figure 5:**
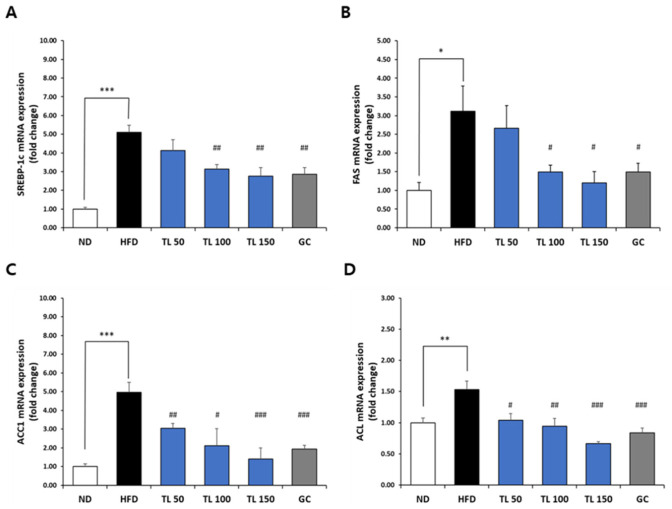
Assessment of lipogenic marker expression in epididymal adipose tissue from mice with HFD-induced obesity. Transcript levels of SREBP-1c (**A**), FAS (**B**), ACC1 (**C**), and ACL (**D**) were quantified using real-time quantitative PCR with specific primer sets. Expression values were normalized to the housekeeping gene GAPDH and presented as fold differences relative to the ND group. Data are presented as means ± SD (*n* = 7 mice per group). * *p* < 0.05, ** *p* < 0.01, and *** *p* < 0.001 denote statistical significance relative to the ND group. ^#^ *p* < 0.05, ^##^ *p* < 0.01, and ^###^ *p* < 0.001 denote statistical significance relative to the HFD group. ND: normal diet; HFD: high-fat diet; TL50: HFD supplemented with TL powder (50 mg/kg BW); TL100: HFD supplemented with TL powder (100 mg/kg BW); TL150: HFD supplemented with TL powder (150 mg/kg BW); GC: HFD supplemented with *Garcinia cambogia* extract (200 mg/kg BW).

**Figure 6:**
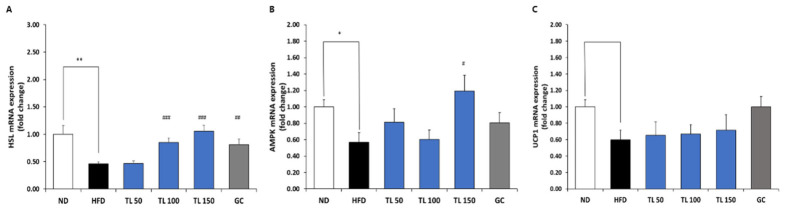
Assessment of lipolysis-, fatty acid oxidation-, and thermogenesis-related marker expression in epididymal adipose tissue from mice with HFD-induced obesity. Transcript levels of HSL (lipolysis) (**A**), AMPK (fatty acid oxidation) (**B**), and UCP1 (thermogenesis) (**C**) were quantified using real-time quantitative PCR with specific primer sets. Expression values were normalized to the housekeeping gene GAPDH and presented as fold differences relative to the ND group. Data are presented as means ± SD (*n* = 7 mice per group). * *p* < 0.05 and ** *p* < 0.01 denote statistical significance relative to the ND group. ^#^ *p* < 0.05, ^##^ *p* < 0.01, and ^###^ *p* < 0.001 denote statistical significance relative to the HFD group. ND: normal diet; HFD: high-fat diet; TL50: HFD supplemented with TL powder (50 mg/kg BW); TL100: HFD supplemented with TL powder (100 mg/kg BW); TL150: HFD supplemented with TL powder (150 mg/kg BW); GC: HFD supplemented with *Garcinia cambogia* extract (200 mg/kg BW).

Gene expression profiles associated with adipocyte differentiation and lipid metabolic pathways were examined in epididymal adipose tissue (Figure 4, Figure 5 and Figure 6). In comparison with the ND group, HFD feeding resulted in a significant elevation of mRNA levels related to adipogenesis including PPARγ, C/EBPα, and aP2 (Figure 4), as well as lipogenesis, including SREBP-1c, FAS, ACC1, and ACL (Figure 5). TL powder supplementation significantly attenuated the HFD-induced upregulation of these genes. Overall, TL-treated groups showed lower expression levels of adipogenic and lipogenic markers than the HFD control group.

To investigate how TL powder influences lipolysis, fatty acid oxidation, and thermogenesis in obese mice, the mRNA expression levels of hormone-sensitive lipase (HSL), AMP-activated protein kinase (AMPK), and uncoupling protein 1 (UCP1) were analyzed, respectively (Figure 6). The expression levels of both, which were suppressed via HFD feeding, were partially restored by TL powder treatment, suggesting enhanced lipolysis and fatty acid oxidation and improved lipid turnover in adipose tissue. In particular, UCP1 mRNA expression in epididymal adipose tissue was substantially lower in abundance compared with other target genes. While not statistically significant, HFD feeding was associated with a downward trend in UCP1 expression, which was partially restored by TL powder supplementation.

## 3. Discussion

In this study, oral administration of TL powder significantly attenuated the body weight gain induced by HFD and effectively reduced the weights of both adipose tissue and liver. These physiological benefits coincide with a significant reduction in adipocyte enlargement and hepatic fat deposition. Such results are consistent with previously established findings that HFD-fed C57BL/6 mice develop substantial increases in fat mass and fatty liver phenotypes, which are widely accepted models for evaluating potential anti-obesity agents.

**Figure 7:**
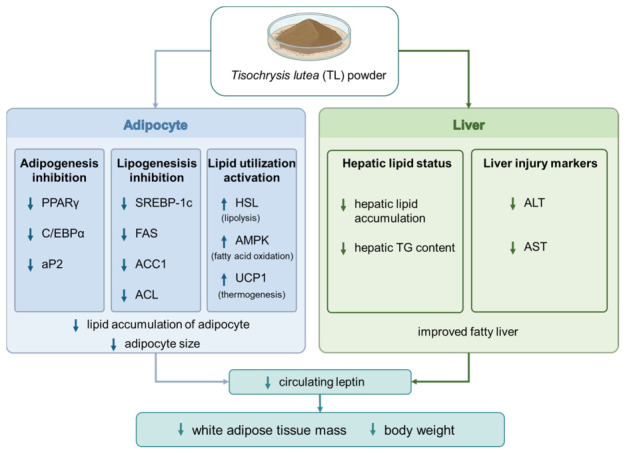
Schematic representation of the molecular mechanisms underlying the anti-obesity effects of TL powder via regulation of adipogenesis and lipid metabolism. In adipocytes, TL powder reduces lipid accumulation by inhibiting adipogenesis and lipogenesis through downregulation of key factors and simultaneously activating lipid utilization via increased HSL, AMPK, and UCP1. In the liver, TL powder decreases fat accumulation and lowers liver injury markers ALT and AST, improving liver health. Overall, TL powder enhances lipid metabolism and protects liver function by reducing fat storage and promoting fat breakdown. ↑: upregulation; ↓: downregulation.

At the molecular level, the downregulation of adipogenic and lipogenic gene expression, including PPARγ, C/EBPα, aP2, SREBP-1c, FAS, ACC1, and ACL, and the partial restoration of hormone-sensitive lipase (HSL) expression upon TL powder supplementation suggest that its anti-obesity effects are mediated by suppressing lipid storage pathways while enhancing lipid turnover within adipose tissue (Figure 7). Moreover, TL powder restored fatty acid oxidation (AMPK), which is downregulated by obesity. These genes and enzymes play pivotal roles as central regulators in adipocyte differentiation and lipid synthesis, and they are commonly used mechanistic endpoints in studies employing HFD-induced obesity mouse models [2,3,34,35,36,37,38,39]. Correspondingly, improvements in serum triglyceride levels and liver enzyme markers (ALT and AST) further support the concept that anti-obesity interventions often act through the adipose–liver axis by reducing ectopic lipid deposition and mitigating metabolic stress [40,41,42].

In particular, the results showing that UCP-1 expression in white adipose tissue is altered by TL are of special significance. UCP-1 is expressed in epididymal white adipose tissue (Epi-WAT) due to the formation of beige adipocytes, indicating that white fat acquires thermogenic function to inhibit fat accumulation. This increase in UCP-1 expression enhances energy expenditure, playing a crucial role in obesity prevention and weight loss. Therefore, maintaining UCP-1 activation by TL is one of the key factors in anti-obesity effects [20,21,22].

Interestingly, the blood glucose data revealed a non-linear dose–response pattern. While TL50 and TL100 showed a trend toward reduced blood glucose compared to the HFD group, the highest dose (TL150) did not yield additional hypoglycemic benefits. This plateau may reflect a U-shaped dose–response commonly observed with natural bioactives [43]. Mechanistically, the robust enhancement of fatty acid oxidation at the highest dose, evidenced by AMPK activation, might shift peripheral energy substrate utilization heavily toward lipids. This shift can lead to a relative sparing of glucose uptake [44], indicating that TL primarily targets lipid remodeling rather than direct glycemic control. Furthermore, the positive control (GC) exhibited elevated blood glucose levels higher than those of the HFD group. This can be explained by the mechanism of hydroxycitric acid (HCA) in GC, which strongly inhibits ATP-citrate lyase, thereby blocking de novo lipogenesis. In a high-fat and energy-surplus state, preventing the conversion of carbohydrates to lipids can paradoxically cause glucose to remain in the blood circulation [45], highlighting the distinct, lipid-centric regulatory mechanisms of TL powder compared to GC.

In HFD-induced obese rodent models, serum total cholesterol levels are typically elevated compared with those of control animals, accompanied by increases in both LDL and HDL cholesterol levels. This phenomenon is commonly observed in HFD-induced rodent models [46,47]. Although HDL cholesterol is generally reduced in human dyslipidemia, the present study showed increased HDL levels in the HFD group. This apparent discrepancy can be attributed to fundamental species-specific differences in lipoprotein metabolism between humans and rodents. In particular, rodents inherently exhibit higher HDL cholesterol levels and lack cholesteryl ester transfer protein (CETP), which plays a key role in lipid exchange among lipoproteins in humans. Consequently, HFD feeding in rodents often results in concurrent increases in both LDL and HDL cholesterol levels [48].

In the experimental group that consumed TL, there were changes in intracellular signaling molecules related to lipid breakdown, but a decrease in feed intake was also observed compared with the control group without TL. Although no direct effects of TL are known, results have been reported that feed intake decreases due to satiety in experimental animals fed microalgae such as Spirulina or Chlorella [49,50]. Therefore, future research is needed to determine whether the anti-obesity effects of TL are related to satiety, and it is considered necessary to investigate the mechanisms by which satiety occurs.

TL powder was formulated as a standardized microalgal ingredient containing fucoxanthin and docosahexaenoic acid (DHA) as key functional and marker compounds, aligning well with the emerging trend of microalgae-based functional foods and nutraceuticals targeting metabolic health improvements [10,11,12,13,51,52]. A plausible biological basis for the observed effects of TL powder lies in the combined actions of fucoxanthin and DHA. Fucoxanthin has demonstrated consistent in vivo anti-obesity activities, including reductions in adiposity, improvements in plasma lipid profiles, and regulation of adipose tissue thermogenesis and hepatic lipid metabolism, particularly in HFD-fed mice [20,21,22,23,24,25,26]. Recent investigations have also highlighted how optimizing fucoxanthin bioavailability, for example via specialized delivery systems, can enhance its anti-obesity efficacy, underscoring translational potential [25]. Parallel to this, DHA is widely recognized for its modulatory effects on lipid metabolism and inflammatory cascades. Mechanistic studies in adipocyte models further suggest that DHA can inhibit adipogenic programs through suppression of PPARγ signaling under specific differentiation contexts [30,31,32,33]. According to the results of this study, the dual-marker standardized TL powder appears to exhibit complementary effects across multiple pathways related to fat storage, lipogenesis, and systemic lipid processing, suggesting that it is a promising candidate for a marine-derived functional ingredient for the treatment of obesity. Even from a developmental perspective, comparative analyses have indicated that complex microalgal matrices may exert multifaceted biological activities beyond single-compound effects, thereby justifying the focus on whole-biomass functional ingredients [53].

Despite these encouraging results, the study has several limitations. Major limitations of this study include the absence of metabolic function tests and inflammation assessments, as well as the fact that the experimental model consisted solely of males. Furthermore, due to the absence of experimental groups treated with pure fucoxanthin or DHA alone at matched concentrations, the suggested complementary or combined effects of these markers require further validation. It is also possible that other bioactive substances in the TL matrix, such as EPA and abundant fatty acids (e.g., oleic, linoleic, and myristic acids) (Table 1), contributed to the observed results, suggesting that the benefits arise from the holistic impact of the whole microalgal biomass. Therefore, the proposed synergistic effect remains a hypothesis and validating it through single-component control experiments is considered a task that needs to be addressed in future research. Moreover, the current focus on transcriptional markers provides valuable mechanistic insights, but further validation at the protein expression level and pathway-specific functional assays would strengthen causal interpretations and improve alignment with the mechanistic effects reported for fucoxanthin-containing interventions [23,24,25,27,28,29]. Additionally, quantification of circulating and tissue concentrations of fucoxanthin metabolites, such as fucoxanthinol, in future studies would enable clearer exposure–response relationships, as suggested by prior human studies and clinical trials on fucoxanthin supplementation [50,53,54]. Moreover, considering the increasing interest in microalgae–gut microbiota interactions in obesity, comprehensive analyses including microbiome profiling and bile acid metabolomics could elucidate systemic mechanisms underlying TL powder’s beneficial effects [11,13,55,56].

## 4. Materials and Methods

### 4.1. Materials

*Tisochrysis lutea* (TL) powder was supplied by Microalgae Ask Us Co., Ltd. (Gangneung, Republic of Korea), and a voucher specimen of the strain was deposited at the Korean Collection for Type Cultures (KCTC, Accession No. AG60897). Garcinia cambogia extract (GC), standardized to hydroxycitric acid (61.70%), was obtained from Mirae Biotech, Inc. (Pocheon, Republic of Korea). Male C57BL/6N mice were obtained from DooYeol Biotech (Seoul, Republic of Korea), and all diets (normal-diet D12450B and high-fat-diet D12492) were purchased from Research Diets Inc. (New Brunswick, NJ, USA).

All analytical standards, chemicals, and solvents used in this study were purchased from Sigma-Aldrich (St. Louis, MO, USA). For biochemical analyses, the leptin ELISA kit and hepatic TG assay kit were obtained from R&D Systems (Minneapolis, MN, USA) and Asan Pharmaceutical (Seoul, Republic of Korea), respectively. Serum separator tubes were sourced from Becton Dickinson (Franklin Lakes, NJ, USA). For gene expression analysis, the RNeasy Mini Kit was purchased from Qiagen (Hilden, Germany), and the PrimeScript™ RT Reagent Kit and TB Green^®^ Premix Ex Taq™ II were purchased from TaKaRa (Shiga, Japan).

### 4.2. Sample Preparation and Characterization of Tisochrysis lutea Powder

The microalgal biomass was cultivated under controlled photoautotrophic conditions. The TL powder was produced as a commercially standardized raw material for functional food applications. It was strictly standardized based on its fucoxanthin and docosahexaenoic acid (DHA) contents to ensure batch-to-batch consistency. The TL powder used in animal experiments complied with the quality specifications established under the Korean Ministry of Food and Drug Safety (MFDS) approval for individually recognized functional food ingredients, including criteria for heavy metals and microbiological safety. The chemical composition of the TL powder was characterized by analyzing fucoxanthin and DHA contents. Fucoxanthin was quantified using high-performance liquid chromatography (HPLC), and DHA content was determined using gas chromatography (GC) following fatty acid methyl ester derivatization, as described below.

#### 4.2.1. Lipid Extraction from TL Powder

For lipid extraction, the method described in the Korean MFDS Food Standards and Specifications [57] was employed with minor adjustments. Briefly, 200 mg of TL powder was combined with 50 mg of pyrogallol in 3 mL of ethanol, along with 1 mL of undecanoic acid methyl ester as an internal standard. After the addition of 8.3 M HCl (10 mL), the mixture underwent thermal hydrolysis at 80 °C for 90 min. Following cooling, the lipids were partitioned twice into a 1:1 (*v*/*v*) blend of diethyl ether and petroleum ether. The resulting extracts were pooled and evaporated to a viscosity using nitrogen gas at 40 °C.

#### 4.2.2. Derivatization of Fatty Acids into Methyl Esters

Fatty acid methyl esters (FAMEs) were prepared by reacting the lipid extract with 3 mL of 7% boron trifluoride–methanol and 1 mL of toluene. The mixture was vigorously vortexed and heated at 100 °C for 90 min in a sealed vessel with shaking every 30 min to ensure complete methylation. After cooling to room temperature, the reaction was quenched with 8 mL of distilled water. Subsequently, 1 mL of n-hexane and 1 g of anhydrous sodium sulfate were added to facilitate phase separation. The mixture was centrifuged at 2000 rpm for 20 min, and the upper organic phase was collected, dried over anhydrous sodium sulfate, and transferred to GC vials for analysis. The derivatization procedure followed the MFDS Food Standards [57].

#### 4.2.3. GC-FID Analysis of Fatty Acid Methyl Esters (FAMEs)

Fatty acid methyl ester analysis was performed using an Agilent 8890 gas chromatograph (Agilent Technologies, Santa Clara, CA, USA) equipped with a flame ionization detector. Separation was achieved on an SP-2560 fused silica capillary column (100 m × 0.25 mm internal diameter, 0.20 µm film thickness) (Supelco, Bellefonte, PA, USA). The oven temperature was initially held at 100 °C for 4 min, then increased to 240 °C at a rate of 3 °C/min, and maintained at 240 °C for 15 min. Helium was used as the carrier gas at a constant flow rate of 0.85 mL/min. The injector and detector temperatures were set at 225 °C and 285 °C, respectively, with a split ratio of 200:1 and an injection volume of 2 µL. Individual fatty acid methyl esters were identified by comparing their retention times with those of authentic reference standards. The analytical procedure followed the Korean MFDS Food Standards [57].

#### 4.2.4. Determination of Fucoxanthin Content via HPLC

Fucoxanthin was analyzed using an Agilent 1260 high-performance liquid chromatography system (Agilent Technologies, Santa Clara, CA, USA) equipped with a CAPCELL PAK C18 MG II column (250 mm × 4.6 mm, 5 µm particle size) (Osaka Soda Co., Osaka, Japan). For sample preparation, TL powder (5–10 mg) was finely ground and extracted with 80% ethanol to obtain a final concentration of 1 mg/mL. The mixture was vortexed and subjected to ultrasonic extraction for 1 h, with intermittent mixing every 10–15 min. After extraction, the solution was filtered through a syringe filter, and the filtrate was used for HPLC analysis.

The analysis was performed according to the method described in [24]. The mobile phase consisted of acetonitrile (A) and water (B) at a flow rate of 1.0 mL/min. The gradient program was as follows: 0–8 min, 90:10 to 100:0 (A:B); 8–11 min, 100:0 (A:B); and 11–16 min, 100:0 to 80:20 (A:B). Detection was carried out at 450 nm. Fucoxanthin was quantified using an external standard calibration curve constructed at concentrations of 1, 5, 10, 50, 100, and 200 µg/mL, using a fucoxanthin analytical standard.

### 4.3. In Vivo Experiments

All experimental procedures involving animals complied with institutional standards for laboratory animal care and were authorized by the Institutional Animal Care and Use Committee (IACUC) of Samyook University (Approval No. SYUIACUC 2025-008). Sixty male C57BL/6N mice (5 weeks old) were obtained from DooYeol Biotech and housed under specific pathogen-free conditions.

After a 1-week acclimation period under controlled temperature (23 ± 1 °C), relative humidity (50 ± 5%), and a 12 h light/dark cycle, with unrestricted access to food and water, animals were allocated randomly into dietary groups. Ten mice received a normal diet (ND) (D12450B, Research Diets Inc., New Brunswick, NJ, USA), whereas fifty mice were provided a high-fat diet (HFD) (D12492, Research Diets Inc.) for 8 weeks to establish obesity. The HFD supplied 5.1 kcal/g (60% fat, 20% carbohydrate, 20% protein), while the ND diet provided 3.7 kcal/g (10% fat, 70% carbohydrate, 20% protein).

Following obesity induction, HFD-fed mice that doubled their body weight were further divided into five groups (n = 10 each): HFD control, TL50 (TL powder 50 mg/kg body weight), TL100 (100 mg/kg body weight), TL150 (150 mg/kg body weight), and GC (*Garcinia cambogia* extract 200 mg/kg body weight, positive control). The dosage of TL powder was determined based on fucoxanthin doses reported in previous anti-obesity animal studies [20,58], and the GC dosage was also selected with reference to a previous study [59]. The number of animals used for statistical analysis was likewise determined based on these prior studies [20,58,59]. GC is a well-known ingredient in the body fat–reduction market. GC contains hydroxycitric acid (HCA), which inhibits lipogenesis by blocking ATP-citrate lyase (ACL). It also suppresses appetite by increasing serotonin and may promote fatty acid oxidation, thereby contributing to weight loss [45]. These combined mechanisms support the use of GC as a positive control for anti-obesity effects in this study.

TL powder and GC, dissolved in sterile distilled water, were administered orally by gavage once daily for an additional 8 weeks. An equal volume of sterile distilled water was administered orally to mice in the ND and HFD groups simultaneously. Body mass and food consumption were monitored on a weekly basis throughout the study period.

### 4.4. Collection of Blood and Tissue

Prior to sacrifice, experimental animals were anesthetized with tribromoethanol diluted in tertiary amyl alcohol. Blood samples were collected via retro-orbital puncture and transferred into serum separator tubes. The samples were allowed to clot at room temperature for 30 min and subsequently centrifuged at 5000 rpm for 10 min to obtain serum. The separated serum was stored at −70 °C until further analysis.

Following blood collection, animals were euthanized, and the liver and white adipose tissue depots, including epididymal, visceral, retroperitoneal, and inguinal fat, were carefully excised. The tissues were rinsed with cold physiological saline, gently blotted dry with filter paper, weighed, and immediately stored at −70 °C for further analysis. A portion of the epididymal adipose tissue was used for total RNA extraction followed by real-time RT-PCR analysis. Part of the liver tissue was fixed in 4% paraformaldehyde (PFA), embedded in paraffin, and subjected to histological staining. The remaining tissues were stored at −70 °C for further analysis.

### 4.5. Biochemical Analysis of Serum

Serum samples were subjected to biochemical analysis to determine concentrations of glucose, triglycerides (TGs), total cholesterol (TC), low-density lipoprotein cholesterol (LDL-C), high-density lipoprotein cholesterol (HDL-C), aspartate aminotransferase (AST), alanine aminotransferase (ALT), creatinine, and blood urea nitrogen (BUN) using an automated clinical chemistry analyzer (KoneLab 20 XT, Thermo Fisher Scientific, Waltham, MA, USA). Circulating leptin levels were quantified using a commercially available enzyme-linked immunosorbent assay (ELISA) kit (R&D Systems, Minneapolis, MN, USA) according to the manufacturer’s protocol [40,41].

### 4.6. Histological Analysis

Epididymal fat and liver tissues were immersed in 4% paraformaldehyde, processed for paraffin embedding, and cut into 5 μm-thick sections [23]. The sections were stained with hematoxylin and eosin (H&E) to examine tissue morphology. Microscopic images were obtained using a light microscope, and adipocyte size in epididymal adipose tissue was quantified from H&E-stained sections with the AxioVision 4.8 image analysis software (Carl Zeiss, Oberkochen, Germany). For each animal, five independent tissue sections were prepared, and five randomly selected, non-overlapping microscopic fields per section were analyzed to reduce sampling bias. All measurements were performed in a blinded manner to ensure objectivity and minimize observer bias.

### 4.7. Quantitative Real-Time PCR Analysis

Total RNA was isolated from epididymal adipose tissue using the RNeasy Mini Kit (Qiagen, Hilden, Germany) according to the supplier’s protocol. RNA yield and purity were determined spectrophotometrically (Epoch™, BioTek Instruments, Winooski, VT, USA). First-strand cDNA was synthesized from 250 ng of total RNA using the PrimeScript™ RT Reagent Kit (TaKara Bio Inc., Shiga, Japan).

Real-time quantitative PCR was performed using TB Green^®^ Premix Ex Taq™ II (TaKara Bio Inc., Shiga, Japan) and the primer sets listed in Table 5. Transcripts associated with adipocyte differentiation and lipid metabolism were quantified. Expression data were normalized to the reference gene GAPDH, and relative mRNA levels were calculated using the 2^−∆∆Ct^ method [2,3].

### 4.8. Determination of Hepatic TG Content

Liver samples were homogenized, and total lipids were isolated according to a modified Folch extraction procedure. TG concentrations in the hepatic extracts were quantified using a commercially available enzymatic assay kit [41,42]. The results are reported as milligrams of TG per gram of liver tissue as well as total milligrams per entire liver.

### 4.9. Statistical Analysis

Statistical evaluations were conducted using GraphPad Prism (version 10.5.0, GraphPad Software, San Diego, CA, USA). Results are expressed as mean ± standard deviation (SD), with n = 10 per group for animal studies and n = 7 per group for gene expression analyses. Differences among groups were analyzed via one-way analysis of variance (ANOVA). Prior to performing the statistical analyses, the normality of the data distribution was assessed using the Shapiro–Wilk test. Based on these assumptions, ANOVA was conducted. Statistical thresholds were defined as * *p* < 0.05, ** *p* < 0.01, and *** *p* < 0.001 versus the ND group, and ^#^ *p* < 0.05, ^##^ *p* < 0.01, and ^###^ *p* < 0.001 versus the HFD group.

## 5. Conclusions

*Tisochrysis lutea* powder, standardized for fucoxanthin (12.18 ± 0.21 mg/g DW) and DHA (16.03 ± 0.49 mg/g DW) content, effectively alleviates diet-induced obesity by suppressing adipogenesis and lipid synthesis while improving lipid metabolism in adipose tissue and liver. This study highlights the novelty of utilizing the whole microalgal biomass rather than extracts, providing in vivo evidence supporting the use of TL powder as a functional microalgal ingredient for obesity prevention and metabolic health improvement. Furthermore, we believe that translational applications would be feasible with additional clinical study results.

## Figures and Tables

**Table 1:** Fatty acid profile of TL powder quantified via GC–FID and expressed as mg per 100 g of dry weight (DW).

Peak No.	Name	Nomenclature	Contents (mg/100 g)
1	Butyric acid	C4:0	0.0
2	Caproic acid	C6:0	0.0
3	Caprylic acid	C8:0	0.0
4	Capric acid	C10:0	0.0
5	Undecanoic acid	C11:0	0.0
6	Lauric acid	C12:0	100.7 ± 17.9
7	Tridecanoic acid	C13:0	5.2 ± 0.2
8	Myristic acid	C14:0	2391.2 ± 307.5
9	Myristoleic acid	C14:1	104.8 ± 18.6
10	Pentadecylic acid	C15:0	81.5 ± 5.9
11	Cis-Pentadecenoic acid	C15:1	0.0
12	Palmitic acid	C16:0	1665.4 ± 185.9
13	Palmitoleic acid	C16:1n-7	1302.9 ± 161.8
14	Heptadecanoic acid	C17:0	43.8 ± 40.8
15	Cis-Heptadecenoic acid	C17:1	51.8 ± 12.0
16	Stearic acid	C18:0	28.0 ± 12.9
17	Trans-Oleic acid	C18:1-trans	0.0
18	Oleic acid	C18:1n-9	2403.2 ± 474.5
19	Trans-Linoleic acid	C18:2-trans	39.6 ± 12.2
20	Linoleic acid	C18:2n-6	1736.2 ± 519.7
21	Arachidic acid	C20:0	23.0 ± 3.7
22	Gamma-linolenic acid (GLA)	C18:3n-6	157.4 ± 43.2
23	Cis-11-eicosenoic acid	C20:1n-9	3.8 ± 0.6
24	Alpha-linolenic acid (ALA)	C18:3n-3	1834.0 ± 243.9
25	Heneicosanoic acid	C21:0	4.1 ± 1.5
26	Eicosadienoic acid	C20:2n-6	2639.6 ± 507.0
27	Behenic acid	C22:0	72.7 ± 17.6
28	Dihomo-γ-linolenic acid	C20:3n-6	0.0
29	Cis-13-docosenoic acid	C22:1n-9	225.6 ± 26.2
30	Eicosatrienoic acid	C20:3n-3	19.0 ± 3.9
31	Tricosanoic acid	C23:0	0.0
32	Arachidonic acid (AA)	C20:4n-6	33.9 ± 14.7
33	Docosadienoic acid	C22:2n-6	21.0 ± 5.5
34	Lignoceric acid	C24:0	24.8 ± 17.4
35	Eicosapentaenoic acid (EPA)	C20:5n-3	76.1 ± 61.4
36	Nervonic acid	C24:1n-9	12.8 ± 1.5
37	Docosahexaenoic acid (DHA)	C22:6n-3	1603.0 ± 48.5
	Total		16,705.2 ± 699.7

**Table 2:** Influence of TL powder administration on body weight and feed efficiency in mice subjected to a high-fat diet.

Parameters	ND	HFD-Induced Obese Mice				
		HFD	TL 50	TL 100	TL 150	GC
Initial body weight (g)	20.3 ± 0.4	20.9 ± 0.3	20.5 ± 0.3	20.8 ± 0.2	20.8 ± 0.2	20.9 ± 0.3
Final body weight (g)	29.4 ± 0.5	41.0 ± 1.0 ***	32.6 ± 0.5 ^###^	30.6 ± 0.5 ^###^	28.2 ± 0.8 ^###^	33.1 ± 0.7 ^###^
Weight gain ^1^ (g)	9.0 ± 0.6	20.1 ± 0.8 ***	12.0 ± 0.4 ^###^	9.8 ± 0.5 ^###^	7.4 ± 0.7 ^###^	11.1 ± 0.4 ^###^
Food intake (g/day)	3.08 ± 0.06	2.85 ± 0.06 **	2.40 ± 0.03 ^###^	2.32 ± 0.03 ^###^	2.20 ± 0.05 ^###^	2.40 ± 0.05 ^###^
FER ^2^	0.054 ± 0.004	0.127 ± 0.004 ***	0.091 ± 0.003 ^###^	0.076 ± 0.003 ^###^	0.060 ± 0.005 ^###^	0.093 ± 0.005 ^###^

^1^ Weight gain was calculated as the difference between final and initial body weight. ^2^ Feed efficiency ratio (FER) was calculated as FER = (weight gain (g)/cumulative food intake (g)) × 100. Values are expressed as means ± standard deviations (SDs), with *n* = 10 mice per group. ** *p* < 0.01 and *** *p* < 0.001 denote statistical significance relative to the ND group. ^###^ *p* < 0.001 denotes statistical significance relative to the HFD group. ND: normal diet; HFD: high-fat diet; TL50: HFD supplemented with TL powder (50 mg/kg BW); TL100: HFD supplemented with TL powder (100 mg/kg BW); TL150: HFD supplemented with TL powder (150 mg/kg BW); GC: HFD supplemented with *Garcinia cambogia* extract (200 mg/kg BW).

**Table 3:** Influence of TL powder administration on white adipose tissue and liver weights in HFD-induced obesity.

Parameters	ND	HFD-Induced Obese Mice				
		HFD	TL50	TL100	TL150	GC
White adipose tissue (g)						
Total weight	1.457 ± 0.160	4.89 6± 0.120 ***	3.641 ± 0.161 ^###^	2.977 ± 0.109 ^###^	2.281 ± 0.246 ^###^	3.654 ± 0.199 ^###^
Epididymal fat	0.692 ± 0.065	2.303 ± 0.079 ***	1.745 ± 0.077 ^###^	1.416 ± 0.070 ^###^	1.065 ± 0.111 ^###^	1.787 ± 0.103 ^###^
Retroperitoneal fat	0.267 ± 0.040	0.972 ± 0.045 ***	0.731 ± 0.039 ^###^	0.590 ± 0.037 ^###^	0.452 ± 0.055 ^###^	0.704 ± 0.037 ^###^
Mesenteric fat	0.255 ± 0.041	0.802 ± 0.084 ***	0.483 ± 0.055 ^##^	0.423 ± 0.038 ^###^	0.337 ± 0.045 ^###^	0.481 ± 0.062 ^##^
Inguinal fat	0.244 ± 0.035	0.819 ± 0.077 ***	0.681 ± 0.040	0.549 ± 0.046 ^##^	0.428 ± 0.059 ^###^	0.683 ± 0.060
Liver (g)	1.053 ± 0.024	1.301 ± 0.084 *	0.939 ± 0.020 ^###^	0.872 ± 0.015 ^###^	0.804 ± 0.032 ^###^	0.934 ± 0.032 ^###^

Values are expressed as means ± standard deviations (SDs), with *n* = 10 mice per group. * *p* < 0.05 and *** *p* < 0.001 denote statistical significance relative to the ND group. ^##^ *p* < 0.01 and ^###^ *p* < 0.001 denote statistical significance relative to the HFD group. ND: normal diet; HFD: high-fat diet; TL50: HFD supplemented with TL powder (50 mg/kg BW); TL100: HFD supplemented with TL powder (100 mg/kg BW); TL150: HFD supplemented with TL powder (150 mg/kg BW); GC: HFD supplemented with *Garcinia cambogia* extract (200 mg/kg BW).

**Table 4:** Influence of TL powder administration on serum biochemical parameters in HFD-induced obesity.

Parameters	ND	HFD-Induced Obese Mice				
		HFD	TL50	TL100	TL150	GC
Glucose (mg/dL)	186.9 ± 13.6	306.6 ± 15.1 ***	290.0 ± 8.0	283.4 ± 10.3	305.7 ± 12.5	333.1 ± 8.8
TG (mg/dL)	51.3 ± 4.0	76.2 ± 3.9 ***	70.7 ± 3.8	60.3 ± 3.4 ^##^	46.9 ± 3.8 ^###^	55.4 ± 3.0 ^##^
Total cholesterol (mg/dL)	126.1 ± 6.7	164.4 ± 5.7 ***	180.0 ± 6.8	171.7 ± 4.9	158.4 ± 4.5	165.2 ± 5.7
LDL-cholesterol (mg/dL)	27.6 ± 1.5	30.2 ± 1.6	33.7 ± 1.9	33.2 ± 1.5	34.2 ± 2.3	31.6 ± 1.8
HDL-cholesterol (mg/dL)	99.2 ± 5.0	127.2 ± 4.0 ***	137.3 ± 4.9	132.9 ± 4.1	124.3 ± 3.1	131.5 ± 3.6
AST (U/L)	54.5 ± 4.3	79.5 ± 5.5 **	64.0 ± 3.3 ^#^	58.3 ± 2.8 ^##^	48.8 ± 2.6 ^###^	60.7 ± 4.3 ^#^
ALT (U/L)	19.9 ± 1.9	48.3 ± 4.8 ***	23.1 ± 1.9 ^###^	20.1 ± 1.1 ^###^	18.5 ± 1.3 ^###^	22.7 ± 1.4 ^###^
Creatinine (mg/dL)	0.461 ± 0.010	0.436 ± 0.007	0.452 ± 0.005	0.433 ± 0.005	0.455 ± 0.007	0.425 ± 0.008
BUN (mg/dL)	25.9 ± 1.3	26.0 ± 1.8	23.2 ± 0.6	22.1 ± 1.6	25.5 ± 2.4	24.0 ± 1.2
Leptin (ng/mL)	6.25 ± 0.81	39.44 ± 2.74 ***	23.74 ± 1.83 ^###^	17.96 ± 2.06 ^###^	11.78 ± 1.96 ^###^	26.05 ± 2.83 ^###^

Values are expressed as means ± standard deviations (SDs), with *n* = 10 mice per group. ** *p* < 0.01 and *** *p* < 0.001 denote statistical significance relative to the ND group. ^#^ *p* < 0.05, ^##^ *p* < 0.01, and ^###^ *p* < 0.001 denote statistical significance relative to the HFD group. ND: normal diet; HFD: high-fat diet; TL50: HFD supplemented with TL powder (50 mg/kg BW); TL100: HFD supplemented with TL powder (100 mg/kg BW); TL150: HFD supplemented with TL powder (150 mg/kg BW); GC: HFD supplemented with *Garcinia cambogia* extract (200 mg/kg BW).

**Table 5:** Primer sequences employed quantitative real-time PCR analysis of mRNA expression.

Gene	Primer Sequence	
PPARγ	F:	5′-CAAAACACCAGTGTGAATTA-3′
	R:	5′-ACCATGGTAATTTCTTGTGA-3′
C/EBPα	F:	5′-TGGACAAGAACAGCAACGAGTAC-3′
	R:	5′-GCAGTTGCCCATGGCCTTGAC-3′
aP2	F:	5′-GGATTTGGTCACCATCCGGT-3′
	R:	5′-TTCACCTTCCTGTCGTCTGC-3′
SREBP-1c	F:	5′-GCTGCTCAACAGCTGTGGC-3′
	R:	5′-ATGGTAGACAACAGCCGCATC-3′
FAS	F:	5′-AGGGGTCGACCTGGTCCTCA-3′
	R:	5′-GCCATGCCCAGAGGGTGGTT-3′
ACC1	F:	5′-GGAGATGTACGCTGACCGAGAA-3′
	R:	5′-ACCCGACGCATGGTTTTCA-3′
ACL	F:	5′-TGGATGCCACAGCTGACTAC-3′
	R:	5′-GGTTCAGCAAGGTCAGCTTC-3′
HSL	F:	5′-CCGTTCCTGCAGACTCTCTC-3′
	R:	5′-CCACGCAACTCTGGGTCTAT-3′
AMPK	F:	5′-GGTGTACGGAAGGCAAAATGGC-3′
	R:	5′-CAGGATTCTTCCTTCGTACACGC-3′
UCP1	F:	5′-GCTTTGCCTCACTCAGGATTGG-3′
	R:	5′-CCAATGAACACTGCCACACCTC-3′
GAPDH	F:	5′-TGTGTCCGTCGTGGATCTGA-3′
	R:	5′-TTGCTGTTGAAGTCGCAGGAG-3′

PPARγ: peroxisome proliferator-activated receptor gamma; C/EBPα: CCAAT/enhancer-binding protein alpha; aP2: adipocyte protein 2; SREBP-1c: sterol regulatory element binding protein-1c; FAS: fatty acid synthase; ACC1: acetyl-CoA carboxylase 1; ACL: ATP-citrate lyase; HCL: hormone-sensitive lipase; AMPK: 5′-AMP-activated protein kinase; UCP1: uncoupling protein 1; and GAPDH: glyceraldehyde-3-phosphate dehydrogenase.

## Data Availability

The corresponding authors can make any materials available upon request. The aggregate data from the referenced datasets are available from the corresponding authors upon reasonable request.
